# Enhancing rigour in the validation of patient reported outcome measures (PROMs): bridging linguistic and psychometric testing

**DOI:** 10.1186/1477-7525-10-64

**Published:** 2012-06-08

**Authors:** Gwerfyl Roberts, Seren Roberts, Richard Tranter, Rhiannon Whitaker, Emma Bedson, Siobhan Tranter, Delyth Prys, Heledd Owen, Yvonne Sylvestre

**Affiliations:** 1 Centre for Health-Related Research, School of Healthcare Studies, Bangor University, Fron Heulog, Ffriddoedd Road, Bangor, Gwynedd, LL57 2EF, UK; 2 Institute of Medical and Social Care Research, Bangor University, Cambrian House, Wrexham Technology Park, Wrexham, LL13 7YP, UK; 3 North Wales Organisation for Randomised Trials in Health, Bangor University, Y Wern, Normal Site, Bangor, Gwynedd, LL57 2PX, UK; 4 School of Medical Sciences, Bangor University, Brigantia Building, Penrallt Road, Bangor, Gwynedd, LL57 2AS, UK; 5 Betsi Cadwaladr University Health Board, Ysbyty Gwynedd, Penrhosgarnedd, Bangor, Gwynedd, LL57 2PW, UK; 6 Language Technology Unit, Canolfan Bedwyr, Bangor University, Dyfrdwy, College Road, Bangor, Gwynedd, LL57 2PX, UK

**Keywords:** BDI-II, Linguistic validation, Patient reported outcome measures, Psychometric validation, Welsh language

## Abstract

**Background:**

A strong consensus exists for a systematic approach to linguistic validation of patient reported outcome measures (PROMs) and discrete methods for assessing their psychometric properties. Despite the need for robust evidence of the appropriateness of measures, transition from linguistic to psychometric validation is poorly documented or evidenced. This paper demonstrates the importance of linking linguistic and psychometric testing through a purposeful stage which bridges the gap between translation and large-scale validation.

**Findings:**

Evidence is drawn from a study to develop a Welsh language version of the Beck Depression Inventory-II (BDI-II) and investigate its psychometric properties. The BDI-II was translated into Welsh then administered to Welsh-speaking university students (n = 115) and patients with depression (n = 37) concurrent with the English BDI-II, and alongside other established depression and quality of life measures. A Welsh version of the BDI-II was produced that, on administration, showed conceptual equivalence with the original measure; high internal consistency reliability (Cronbach’s alpha = 0.90; 0.96); item homogeneity; adequate correlation with the English BDI-II (r = 0.96; 0.94) and additional measures; and a two-factor structure with one overriding dimension. Nevertheless, in the student sample, the Welsh version showed a significantly lower overall mean than the English (*p* = 0.002); and significant differences in six mean item scores. This prompted a review and refinement of the translated measure.

**Conclusions:**

Exploring potential sources of bias in translated measures represents a critical step in the translation-validation process, which until now has been largely underutilised. This paper offers important findings that inform advanced methods of cross-cultural validation of PROMs.

## Background

Patient reported outcome measures (PROMs) are used increasingly in clinical practice and research where they must be fit for purpose and sensitive to patients’ cultural and linguistic needs [1]. Thus PROMs are required in a range of different languages; and the need to maintain reliability and validity of measures is paramount [2]. Whilst a rigorous multi-step approach to translation is endorsed [3,4], there are no clear recommendations about the early assessment of reliability and validity of translated measures before large-scale testing. We demonstrate the value of undertaking early checks to refine measures. Our case in point is the translation and validation of the Beck Depression Inventory II (BDI-II) [5] for the Welsh language. The measure is widely used both clinically and in research for measuring the severity of depression and response to psychological and medical interventions; and it is one of the PROMS recommended by the Welsh and UK Governments for screening depression in high risk populations in primary care.

The BDI has been translated into numerous languages and is psychometrically robust for use in countries across the world [6-8]. There is, however, no Welsh language version currently available. Here, we report the linguistic and psychometric validation of the Welsh BDI-II and highlight the value of embedding early stage validation within the instrument development phase.

## Methods

### Linguistic validation

Under licence of the publisher and adopting the International Society for Pharmacoeconomics and Outcomes Research (ISPOR) guidelines [3], two independent translators produced a Welsh BDI-II. Reconciliation of these translations into a merged document was undertaken through consensus. This version was then translated back into English by a third independent translator for quality assurance. Comparison between the back translation and original measure highlighted any discrepancies which were revised through discussion and consensus. Eight Welsh-speaking lay respondents (Table 1) were invited to complete the Welsh BDI-II and check their comprehension and interpretation of the draft measure. Remaining discrepancies were identified by comparing these interpretations with the original measure. A final Welsh translation was agreed and subjected to an early exploratory stage of psychometric testing. In line with previous validation of the BDI-II [5], two test groups were identified: (i) a student sample, and (ii) a clinical sample of patients with depression (Table 1).

**Table 1:** Characteristics of the study samples

**Characteristic**		**Cognitive testing**	**Sample**	
			**Student**	**Clinical**
N		8	115	37
Gender, n (%)				
	Female	5 (62.5)	94 (81.7)	22 (59.5)
	Male	3 (37.5)	21 (18.3)	15 (40.5)
Age, n (%)				
	<17 years	1 (12.5)		
	17-24 years	2 (25)	73 (63.5)	1 (2.7)
	25-34 years	2 (25)	18 (15.7)	7 (18.9)
	35-44 years	1 (12.5)	16 (13.9)	13 (35.1)
	45-54 years	2 (25)	7 (6.1)	7 (18.9)
	> 54 years	0 (0)	1 (0.9)	9 (24.3)
Percentage of time Welsh is spoken				
	Range	N/A	5-100	5-100
	Median (IQR)	N/A	90 (60 to 95)	70 (80 to 95)
	Missing		5	6
BDI-II: Welsh				
	Range	N/A	0-35	19-62
	Mean (SD)	N/A	5.1 (5.9)	38.4 (11.9)
	Median (IQR)	N/A	3 (1 to 7)	39 (28.5-46)
	Missing		4	-
BDI-II: English				
	Range	N/A	0-34	15-61
	Mean (SD)	N/A	5.7 (5.5)	37.7 (11.5)
	Median (IQR)	N/A	5 (1.1 to 8)	38 (28.5-47)
	Missing		1	-

### Psychometric testing

In keeping with theoretical propositions [1], the Welsh BDI-II was expected to have (a) a two-factor structure similar to the original model presented, and (b) adequate correlations with other accepted depression scales, and negative correlations with quality of life scales. These hypotheses were tested by (a) performing a confirmatory factor analysis on the student sample data and (b) examining Pearson correlation coefficients between the Welsh BDI-II and other pre-specified measures, including the English BDI-II, for both the clinical and student samples. Further exploratory item level analysis was undertaken to identify potential sources of bias.

### Student sample

Out of 144 bilingual (Welsh/English) university students approached, 115 (80%) consented to participate in the study. Data collection was undertaken during 2009 in a classroom setting, outside teaching hours, where participants were asked to complete the following measures in the order listed:

(a) BDI-II (English) [5]

(b) European Quality of Life-5 Dimensions (EQ-5D) (Welsh) [9]

(c) Hospital Anxiety and Depression Scale (HADS) (English) [10]

(d) Short-Form 12-item Health Survey version 2 (SF-12 v2) (English) [11]

(e) BDI-II (Welsh) [5]

## Clinical sample

A sample of Welsh-speaking patients with depression was recruited to participate in this validation study between 2009 and 2010 through the Folate Augmentation of Treatment - Evaluation for Depression (FolATED) trial [12]. Thirty-seven of 81 (46%) bilingual speakers consented to participate. Consistent with the trial protocol, the following English measures were completed at randomisation (followed by other trial measures):

(a) BDI-II [5]

(b) Researcher-rated Montgomery-Asberg Depression Rating Scale [13]

(c) SF-12 v2 [11]

(d) EQ-5D [9]

For the validation study, participants were also invited to complete the Welsh BDI-II.

Bangor University School of Healthcare Sciences Ethics Committee approved the student study whilst the Multi-centre Research Ethics Committee for Wales approved the patient study through the FolATED trial processes [12]. All data were anonymised and analysed using PASW [14] and AMOS [15] for Windows (version 18.0). All statistical tests were two-sided, and *P*-values of ≤0.05 were considered statistically significant.

## Results

The Welsh BDI-II showed a high level of internal consistency for both student (α = 0.90) and clinical (α = 0.96) samples similar to that reported for the English BDI-II (α = 0.87 student sample; α = 0.92 clinical sample) and by Beck and colleagues (α = 0.93) [5]. The Welsh measure demonstrated a high degree of concurrent and discriminant validity with a positive correlation with HADS (student sample: depression component r = 0.71; anxiety component r = 0.66); and negative correlation with the mental component of SF-12v2 (student sample: r = −0.74; clinical sample: r = −0.71) and EQ-5D (student sample: r = −0.66; clinical sample: r = −0.55). Factor analysis revealed a two factor structure emerging from both samples for each language version; with one overriding depression-related dimension. However, confirmatory factor analysis of the student data revealed that the three indices did not meet the criteria for good fit (GFI = 0.54, AGFI = 0.47, RMR = 0.06).

**Figure 1:**
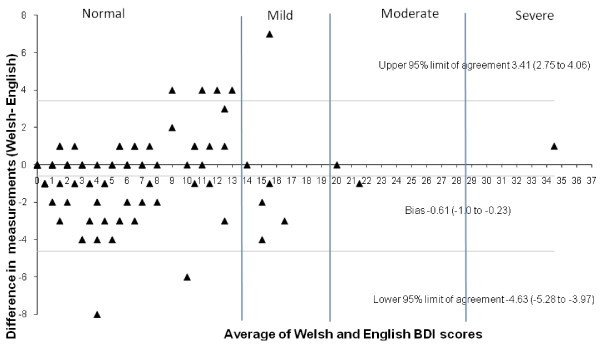
Bland Altman plot for Welsh and English BDI-II (student sample).

The student Welsh BDI-II depression score was highly correlated to the English (r = 0.94), but the overall mean was significantly lower (Welsh M = 5.09, SD = 5.85; English M = 5.70, SD = 5.5), t_110_ = 3.217, *p* = 0.002. The Bland Altman graph [16] (Figure 1) revealed a small but significant bias towards the English BDI-II, showing a slightly higher score than its Welsh comparator; the mean difference (MD) in scores being just over half a point (MD = 0.61, 95% limits of agreement 0.23 to 1.00). The depression score on the Welsh BDI-II was also highly correlated to the English (r = 0.96) within the clinical sample but no statistically significant differences were noted between the mean scores.

Given the evidence of a seemingly biased measure and poorly fitting confirmatory factor analysis for the student sample, further item-level exploration was performed. No differences were found within the clinical sample between mean scores of the Welsh and English BDI-II for the individual items; and there were no indications of asymmetry. However, within the student sample, six items showed statistical significant differences on a paired *t*-test comparing mean scores between the Welsh and English BDI-II. Three of these items also indicated significant asymmetry (Table 2). Close inspection of the three items demonstrating bias revealed potential interpretations that may have led to an underscoring of the item in the Welsh BDI-II (Table 3).

**Table 2:** Item level analysis of the BDI-II (student sample)

**Item**	**Mean**		**S.D.**		**% of participants reporting symptom**		**Item-total correlation**		**Weighted Kappa coefficient**	**Generalised McNemar**
										**asymmetry**
	Welsh	English	Welsh	English	Welsh	English	Welsh	English		*p*-value
1. Sadness	0.05	0.04	0.21	0.21	4.5	4.39	0.48	0.33	0.79	1
2. Pessimism	0.26	0.28	0.46	0.47	27.52	27.19	0.52	0.43	0.76	0.99
3. Past Failure	0.19	0.17	0.48	0.44	14.55	14.04	0.55	0.37	0.76	0.77
4. Loss of Pleasure	0.18	0.18	0.39	0.41	17.27	16.67	0.6	0.36	0.66	0.8
5. Guilty Feelings	0.27	0.32	0.58	0.54	22.02	28.95	0.71	0.57	0.73	0.26
6. Punishment Feelings*	0.02	0.06	0.14	0.24	1.8	6.14	0.36	0.31	0.43	**0.03**
7. Self-Dislike*	0.25	0.37	0.59	0.63	20	28.95	0.59	0.55	0.61	0.3
8. Self - Criticalness*	0.35	0.47	0.62	0.72	29.09	34.21	0.7	0.67	0.74	0.75
9. Suicidal Thoughts*	0.03	0.07	0.17	0.26	2.7	7.02	0.38	0.14	0.53	**0.03**
10. Crying	0.22	0.18	0.54	0.45	18.18	14.91	0.55	0.43	0.82	0.54
11. Agitation	0.21	0.29	0.41	0.46	20.9	28.95	0.6	0.52	0.63	0.07
12. Loss of interest	0.08	0.12	0.28	0.32	8.2	12.28	0.45	0.25	0.6	0.16
13. Indecisiveness	0.33	0.34	0.63	0.67	26.36	25.44	0.62	0.57	0.69	0.63
14. Worthlessness	0.2	0.21	0.51	0.56	14.55	14.91	0.61	0.56	0.66	0.99
15. Loss of Energy*	0.36	0.46	0.48	0.5	35.5	45.61	0.42	0.37	0.66	**0.02**
16. Changes in Sleeping Pattern	0.51	0.55	0.69	0.68	41.8	46.4	0.5	0.5	0.94	0.68
17. Irritability	0.23	0.28	0.42	0.49	22.7	25.44	0.59	0.51	0.63	0.73
18. Changes in Appetite	0.29	0.34	0.55	0.56	24.8	29.8	0.39	0.39	0.9	0.94
19. Concentration Difficulty	0.37	0.38	0.57	0.56	31.82	33.33	0.61	0.62	0.58	0.56
20. Tiredness or Fatigue*	0.44	0.54	0.55	0.58	41.82	50	0.57	0.56	0.72	0.6
21. Loss of interest in Sex	0.23	0.21	0.5	0.49	19.09	18.42	0.28	0.25	0.97	0.99

N = 106 for Welsh BDI-II and 112 for English BDI-II, * = Difference in mean is significant at the .05 level (paired *t*-test).

**Table 3:** Summary of items and potential interpretations which caused bias in the student sample

**Item**	**Score**	**Statement**	**Welsh translation (lit.)**	**Potential interpretation**	**Potential scoring impact**
6.Punishment feelings	0	I don’t feel I am being punished	I don’t feel I am being punished		
	1	I feel I may be punished	^1^Rydw i’n teimlo y *gallwn i gael fy nghosbi* (I feel I **may/can** be punished)	Welsh translation reflects stronger punishment feelings	Underscoring of item in Welsh BDI-II
	2	I expect to be punished	I expect to be punished		
	3	I feel I am being punished	I feel I am being punished		
9.Suicidal thoughts	0	I don’t have any thoughts of killing myself	^*2*^*Dydw i ddim yn meddwl am ladd fy hun***(I am not thinking of** killing myself)	Welsh translation reflects stronger suicidal thoughts	Underscoring of item in Welsh BDI-II
	1	I have thoughts of killing myself but I would not carry them out	^*3*^*Rydw i wedi meddwl am ladd fy hun ond fyddwn i byth yn gwneud* (I have **thought** of killing myself but I would **never do it)**	Welsh translation reflects stronger suicidal thoughts	Underscoring of item in Welsh BDI-II
	2	I would like to kill myself	I would like to kill myself		
	3	I would kill myself if I had the chance	I would kill myself if I had the chance		
15.Loss of energy	0	I have as much energy as ever	I have as much energy as ever		
	1	I have less energy than I used to have	I have less energy than I used to have		
	2	I don’t have enough energy to do very much	^*4*^*Does gen i ddim digon o egni i wneud fawr o ddim* (I don’t have enough energy to do **much of anything)**	Welsh translation reflects greater loss of energy	Underscoring of item in Welsh BDI-II
	3	I don’t have enough energy to do anything	I don’t have enough energy to do anything		

^1^ ‘*Gallwn*’ is a derivative of the auxillary verb ‘*gallu*’ (may/can/be able to). ^2 3^The literal translation of ‘thoughts of killing myself’ is ‘*meddyliau am ladd fy hun’* but the plural form is not a natural expression in Welsh. Hence the adoption of the singular form ‘*meddwl*” (thought). ^4^’Gwneud fawr o ddim’ (lit. do much of anything) is a natural expression in Welsh meaning’do very much’.

## Discussion

We have demonstrated how a thorough and rigorous approach to early validation can inform the refinement of translated outcome measures. Here, we examine the juxtaposition of these two processes (often reported independently in the literature); and discuss the wider implications for a revision of the guidelines and methods of cross-cultural validation of PROMs.

Our results support previous findings on the psychometric properties of the BDI-II, particularly in relation to the two-factor structure [5,7,8,17]; and concurrent validity with other depression and quality of life measures [18-20]. This indicates that the translation and early validation process was relatively successful. Despite the high correlation between the two language versions, the observed poor fit (indicating poor construct validity) and bias led us to explore potential sources of bias and items of concern. This prompted further scrutiny of the translated items to rule out any inaccuracies or misinterpretations, thus providing the opportunity to amend any problematic items. Whilst this step is acknowledged in the literature [4,21], it attracts little attention within current translation and validation guidelines [3,22].

In light of our evidence, it is possible that ambiguities in translation at the lower end of the scale biased response to some items. This interpretation is strengthened as we detected no other subtle dissonances when the remaining items were similarly scrutinised. Moreover, since the student data aggregated to the lower end of the scale, this bias is not observed amongst the clinical sample because the majority reported symptoms of moderate to severe depression. Thus, whilst we acknowledge that our samples were small; our results are suggestive of a potential bias found at the lower end of the scale. A stronger study design involving a qualitative exploration of the students’ interpretations of the discrepant items may well have endorsed this finding.

Whilst this finding led to the refinement of the Welsh BDI-II, it also has several wider implications for instrument translators and developers. Firstly, it draws attention to the need for careful scrutiny in the translation of everyday vocabulary. Secondly, it demonstrates the importance of ensuring that the translated version of a measure is scaled in an equivalent way as the original version. Thirdly, and more importantly, this finding confirms the value of investigating item discrepancies through early exploratory psychometric evaluations of translated measures prior to large-scale, psychometric testing.

### Recommendations

On the basis of our findings, we propose an additional final step (early psychometric testing) to the ISPOR guidelines [3]. This offers a novel, cost-effective approach towards bridging the linguistic and psychometric testing of PROMs that plugs a gap in the current literature and brings the rigour associated with clinical research development to the translation and validation platform.

## Abbreviations

(AGFI), Adjusted Goodness of Fit Index; (AMOS), Analysis of Moment Structures for Windows; (BDI-II), Beck Depression Inventory-II; (EQ-5D), European Quality of Life-5 Dimensions; (FolATED), Folate Augmentation of Treatment - Evaluation for Depression; (GFI), Goodness of Fit Index; (HADS), Hospital Anxiety and Depression Scale; (ISPOR), International Society for Pharmacoeconomics and Outcomes Research; (PROMs), Patient reported outcome measures; (PASW), Predictive Analytic Software; (RMR), Root Mean Square Residual; (SF-12 v2), Short-Form 12-item Health Survey version 2.

## Competing interests

The authors declare that they have no competing interests.

## Authors’ contributions

GR conceptualised and designed the study, acquired and interpreted the data and drafted the manuscript. SR and RT conceptualised and designed the study, acquired and interpreted the data and revised the manuscript. RW supervised the data analysis, interpreted the data and revised the manuscript. EB acquired and interpreted the data and revised the manuscript. ST, DP and HO acquired the data and YS analysed the data. All authors read and approved the final manuscript.

## Authors’ information

GR is director of LLAIS, the Language Awareness Infrastructure Support Service of the National Institute for Social Care and Health Research (NISCHR) Clinical Research Centre in Wales, UK. LLAIS is committed towards developing and validating Welsh language versions of PROMs for the bilingual context of Wales; and establishing the evidence base for best practice in the translation and validation of outcome measures.
